# Hereditary Thrombophilia and thrombotic events in pregnancy: single-center experience

**Published:** 2014

**Authors:** L Coriu, R Ungureanu, R Talmaci, V Uscatescu, M Cirstoiu, D Coriu, E Copaciu

**Affiliations:** * University Emergency Hospital, Bucharest, Romania; ** Fundeni Clinical Institute, Romania

**Keywords:** thrombosis, pregnancy, hereditary thrombophilia

## Abstract

Pregnancy is a normal physiological state that predisposes to thrombosis, determined by hormonal changes in the body. These changes occur in the blood flow (venous stasis), changes in the vascular wall (hypotonia, endothelial lesion) and changes in the coagulation factors (increased levels of factor VII, factor VIII, factor X, von Willebrand factor) and decreased activity levels of natural anticoagulants (protein C, protein S).

In this study, we tried to determine a possible association between thrombosis and inherited thrombophilia in pregnant women.

This is a retrospective study of 151 pregnant women with a history of complicated pregnancy: maternal thrombosis and placental vascular pathology (intrauterine growth restriction, preeclampsia, recurrent pregnancy loss), who were admitted in our hospital during the period January 2010 to July 2014. We performed genetic analyses to detect the factor V Leiden mutation, the G20210A mutation in the prothrombin gene, the C677T mutation and the A1298C mutation in methylenetetrahydrofolate reductase (MTHFR) gene.

The risk of thrombosis in patients with factor V Leiden is 2.66 times higher than the patients negative for this mutation (OR 2.66 95% CI 0.96-7.37 P=0.059). We did not find any statistical association with mutations in the MTHFR gene. Pregnant women with a family history of thrombosis present a 2.18-fold higher risk of thrombosis (OR 2.18 CI 0.9-5.26 P=0.085). Of 151 pregnant women, thrombotic events occurred in 24 patients: deep vein thrombosis, pulmonary embolism, cerebral venous sinus thrombosis and ischemic stroke. The occurrence of thrombotic events was identified in the last trimester of pregnancy, but especially postpartum. Thrombosis in pregnancy is a redoubtable complication requiring an excellent cooperation between the obstetrician and anesthesiologist.

## Introduction

Pregnancy is a normal physiological state that predisposes to thrombosis, a hypercoagulability (thrombophilia), determined by changes in the body, arising from the special hormonal constellation. Such changes occur in blood flow (venous stasis), in the vascular wall (hypotonia, endothelial lesion) and in coagulation factors (increased levels of factor VII, factor VIII, factor X, von Willebrand factor) and decreased activity levels of natural anticoagulants (protein C, protein S). All these changes persist for another 6 weeks post partum [**1**].

In addition, in some cases, over this physiological hypercoagulable state are overlapping consequences of hereditary or acquired “defects” in anticoagulant mechanisms - the hereditary thrombophilia. Hereditary defects related to thrombosis, relatively recently discovered, include the G1691A mutation in the factor V gene, the G20210A mutation in the prothrombin gene, the C677T mutation and the A1298C mutation in methylenetetrahydrofolate reductase (MTHFR) gene, antithrombin III, protein C and protein S deficiency. Among the acquired defects, the most important thing to remember is the antiphospholipid antibody syndrome, but what should not be forgotten are the chronic myeloproliferative syndromes, cancer, paroxysmal nocturnal hemoglobinuria.

Venous thromboembolism (VTE) during pregnancy occurs with an incidence of 1/1000 births [**2**]; with an incidence of VTE of 4.5 times higher in pregnant women compared to non-pregnant. 75-80% of VTE associated pregnancy are deep vein thrombosis (DVT) and 20-25% are pulmonary embolism (EP) [**3**,**4**]. The risk of VTE is higher in the third trimester, but the highest risk is observed postpartum. Thus, the relative risk is 20 times higher in the first 6 weeks postpartum, and 80% of this thrombotic events occur in the first three weeks postpartum [**5**,**6**]. Unlike nonpregnant women who usually develop distal vein thrombosis (DVT), in pregnancy, most thrombotic events are located in the left ileofemural veins [**7**]. This is because of increased venous stasis in the left leg due to the compression of the left iliac vein by the right iliac artery, in addition to compression of the inferior vena cava by the gravid uterus [**8**].

The risk factors in cerebral venous thrombosis (CVT) in adults are the following: hereditary or acquired thrombophilia, oral contraceptives, pregnancy and puerperium, cancer, infection and trauma, with a female predominance of 3:1 [**9**]. As the number of delivery by Caesarean section is increasing every year in many maternity hospitals in our country, what should be noted is that this procedure has been proven to be associated with a 3–12 fold increased risk of ischemic stroke in peripartum and postpartum [**10**,**11**].

This article presents our experience in the diagnosis and treatment of a group of patients with pregnancy-related thrombotic events in which thrombophilic mutations were identified.

## Material and Methods 

151 pregnant women admitted in Bucharest University Emergency Hospital, Department of Obstetrics, with a history of pregnancy complications were studied during January 2010 and July 2014: VTE, intrauterine growth restriction, preeclampsia, recurrent miscarriages.

Inclusion criteria:

• Pregnant

• Thrombotic events and maternal/ placental complications related to pregnancy.

Exclusion criteria:

• Acquired Thrombophilia (e.g. antiphospholipid syndrome)

• Autoimmune Diseases

• Cancer

The diagnosis of thrombosis was suspected on clinical features and confirmed by imaging methods (serial compression ultrasonography of the veins, echocardiography, head MRI, computed tomographic pulmonary angiography).

Laboratory tests: Coagulation tests were performed in the laboratory of Haemostasis, “Fundeni” Center of Hematology and Bone Marrow Transplantation. IL - Instrumentation Laboratory was used: APCR -FV determination was done by using the plasma-based functional clotting assay (cut-off> 2.9); determination of protein C was done by the chromogenic method (normally 70-140%), determination of free Protein S was done by immunological assays: (normally 54-123% female), determination of AT III was done by the chromogenic method (normal 80-120%), determination of D-dimer was done by turbidimetric assay (normal 0- 550 μg / L).

Molecular analysis: Was conducted in the Laboratory of Molecular Biology Hematology, “Fundeni” Center of Hematology and Bone Marrow Transplantation.

2 ml were collected in EDTA venous blood from patients who met the study inclusion criteria. The equipment used for molecular diagnosis was the following: Real Time PCR Platform Light Cycler 480, Termocycler Gene Amp PCR System 9700 Classic, Nano Drop ND-1000 Spectrophotometer, and Electrophoresis Systems. After PCR amplification, mutation identification (G1691A in factor V mutation, G20210A prothrombin gene mutation, C677T/ A1298C mutations in the MTHFR gene) was performed by RFLP, Real Time PCR, and in some cases, direct sequencing.

Statistical analysis: Data obtained from patients were introduced in a database in Excel and were statistically analyzed by using STATA software/ SE11. For quantitative measurements (continuous) the mean, standard deviation was calculated. For qualitative measurements - ordinal and nominal - absolute frequency (number of occurrences) was calculated and the prevalence was expressed as a percentage. For the comparison of qualitative data, Chi square test was used. All the variables collected were statistically analyzed and presented descriptively. Inferential statistical analysis aimed to investigate the relationship between the risk factors and thrombosis. The risk factors investigated were the following: Factor V Leiden mutation genotype, factor II G20210A mutation genotype, MTHFR C677T mutation genotype, MTHFR A1298C mutation genotype, MTHFR C677T/ A1298C heterozygous compound. The association between risk factors and thrombosis was assessed by Chi square test and the actual statistical association was evaluated by calculating the odds ratio (OR) and 95% CI confidence interval by using the univariate logistic regression. All the tests used were bilateral. The threshold of statistical significance was P ≤0.05.

This study was conducted according to the local and national ethical rules.

## Results and Discussion

151 patients with mean age 32.4 ± 4.4 years were included in the study. The minimum age was 22 years and the maximum 46 years. Most patients were between 30-35 years old (**Table 1**).

**Table 1:** Distribution of patients according to the age group

Age group	N	%
Under 29 years old	40	27.4
30-35 years old	78	50
Over 36 years old	33	22.6
Total	146	100

It was found that these pregnant women showed a family history of VTE, myocardial infarction or stroke in the first-degree relatives, younger than 50 years. The prevalence of this history was 34.4% (52 patients) compared to 65.6% (99 patients) who did not (**Table 2**).

**Table 2:** Family history

	N	prevalence (%)
no history	99	65.6
history	52	34.4
Total	151	100

There is a positive association between thrombosis and the presence of positive family history. The risk of thrombosis in patients with a family history was 2.18 times higher than of those without such a history (OR 2.18 CI 0.9-5.26 P=0.085). The association was not statistically significant, but showed a trend (**Table 3**).

**Table 3:** The association between thrombosis and family history

	Thrombosis					
	Absent		Present		Total	
Family history	N	%	N	%	N	%
Absent	87	57.6	12	8.0	99	65.6
Present	40	26.5	12	7.9	52	34.4
Total	127	84.1	24	15.9	151	100.0
Chi square test – P = 0.080						
Logistic regression	OR		95%CI		P	
	2.18		0.9-5.26		0.085	

Regarding the family history of thrombosis, the most important single risk factor for VTE in pregnancy was thrombophilia [**13**,**14**]. Thrombophilia was present in 20-50% of the women who had VTE during pregnancy or postpartum [**12**]. Both acquired or inherited thrombophilia increase the risk.

The association between the occurrence of thrombosis and thrombophilic mutations was assessed in the study group of 151 pregnant women: FV Leiden mutation, 20210 prothrombin gene mutation, MTHFR gene mutations.

There was a positive association between thrombosis and factor V Leiden. The risk of thrombosis in patients with factor V Leiden (heterozygous or homozygous) was 2.66 times higher than that of the patients negative for this mutation (OR 2.66 95% CI 0.96-7.37 P=0.059). The association was not statistically significant but showed a trend (**Table 4**).

**Table 4:** Association between thrombosis and factor V Leiden

	Thrombosis					
	Absent		Present		Total	
Factor V Leiden	N	%	N	%	N	%
Negative	110	72.9	17	11.3	127	84.1
Heterozygote	17	11.3	5	3.3	22	14.6
Homozygote	0	0.0	2	1.3	2	1.3
Total	127	84.1	24	15.9	151	100.0
Chi square test – P = 0.003						
Logistic regression	OR		95%CI		P	
	2.66		0.96-7.37		0.059	

These results were similar to those of the American College of Obstetricians and Gynecologists (ACOG) - thrombotic risk in patients with FVL homozygous population was 4 times higher, and for heterozygous, FVL was 0.5-1.2 times higher [**15**].

The association between thrombosis and the presence of FII G20210A mutation in the prothrombin gene was negative (OR 0.32 95% CI 0.04-2.58 P = 0.288) (**Table 5**). Unfortunately, the number of cases with this mutation in the study was too small to obtain meaningful data. In literature, the risk was 2.4 in homozygous and heterozygous, 0.5 higher than the normal population, according to ACOG [**15**].

**Table 5:** Association between thrombosis and factor II G20210A

	Thrombosis					
	Absent		Present		Total	
Factor II G20210A	N	%	N	%	N	%
Negative	112	74.2	23	15.2	135	89.4
Heterozygote	15	9.9	1	0.7	16	10.6
Total	127	84.1	24	15.9	151	100.0
Chi square test – P = 0.264						
Logistic regression	OR		95%CI		P	
	0.32		0.04-2.58		0.288	

Similar to literature, there was no positive association (OR 0.69 95% CI 0.28-1.7 P = 0.416) between thrombosis and MTHFR C677T gene mutations (**Table 6**).

**Table 6:** Association between thrombosis and MTHFR C667T

	Thrombosis					
	Absent		Present		Total	
MTHFR C667T	N	%	N	%	N	%
Negative	37	24.5	9	6.0	46	30.5
Heterozygote	70	46.4	9	6.0	79	52.3
Homozygote	20	13.3	6	4.0	26	17.2
Total	127	84.1	24	15.9	151	100.0
Chi square test – P = 0.264						
Logistic regression	OR		95%CI		P	
	0.69		0.28-1.7		0.416	

Also, no association between the presence of thrombosis and compound heterozygous MTHFR C677T/ A1298C (OR 0.63 95% CI 0.23-1.7 P = 0.36) was found (**Table 7**).

**Table 7:** Association between thrombosis and compound heterozygous MTHFR C667T + MTHFR A1298C

	Thrombosis					
	Absent		Present		Total	
Compound heterozygous MTHFR C667T + MTHFR A1298C	N	%	N	%	N	%
Negative	83	55.0	18	11.9	101	66.89
Heterozygote	44	29.1	6	4.0	50	33.11
Total	127	84.1	24	15.9	151	100.0
Chi square test – P = 0.357						
Logistic regression	OR		95%CI		P	
	0.63		0.23-1.7		0.36	

The epidemiological studies on large populations (Leiden Thrombophilia Study, the Physicians' Health Study) found no significant increase in risk of VTE in individuals homozygous for the MTHFR mutation. Data published so far about the involvement of MTHFR gene mutation in VTE occurred in pregnancy are less definitive, but there is clearly a trend [**16**,**17**].

**Fig. 1:**
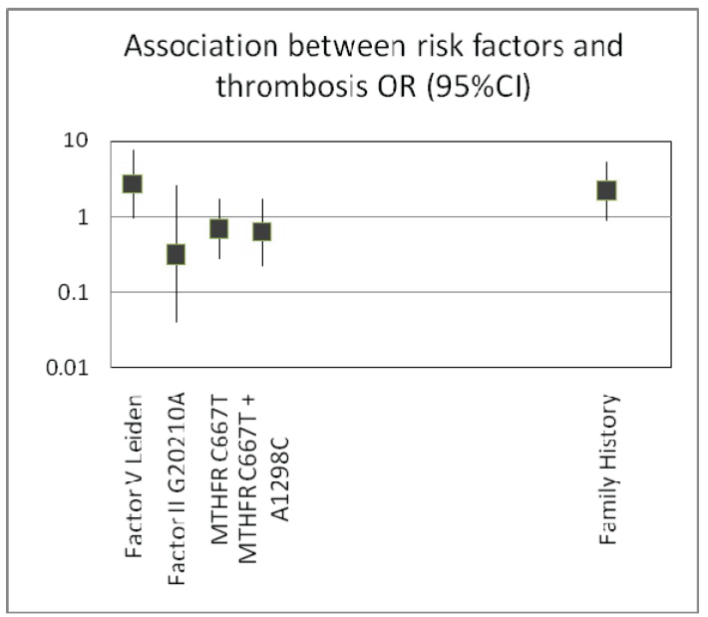
Statistical analysis shows that of all the variables studied, only factor V Leiden and the presence of family history of thrombosis have Odds Ratio greater than 1, so this means that these patients are at risk of thrombosis

The statistical analysis showed that of all the variables studied, only factor V Leiden and the presence of family history of thrombosis have the Odds Ratio greater than 1, so this means that these patients are at risk of thrombosis (**Fig. 1**).

In our group of 151 pregnant women, the thrombotic events occurred in 24 patients (15.9%). Thirteen cases had a deep venous thromboembolism of lower limbs, five cases had pulmonary embolism, three cases had cerebral venous sinuses thrombosis and three cases had cerebral ischemic stroke. Of these, 8.3% occurred in the first trimester of pregnancy (two cases of distal DVT), 16.6% occurred in the second trimester of pregnancy (three cases of DVT distal and one case of longitudinal cerebral sinus thrombosis), 33.3% occurred in the third trimester of pregnancy (three proximal DVT cases, three pulmonary embolisms, one ischemic stroke of the middle cerebral artery, one thrombosis of transverse cerebral sinus). 41.6% of the thrombotic events occurred postpartum, up to two months of puerperium (five cases of DVT, two pulmonary embolisms, one transient ischemic attack of the anterior cerebral artery, one longitudinal sinus thrombosis and one ischemic attack of the middle cerebral artery).

Of the 24 pregnant women with thrombosis, seven cases were positive for FV Leiden mutation; one pregnant woman had G20210A mutation in prothrombin gene; six pregnant women had homozygous C6777T mutation in the MTHFR gene; six pregnant women had compound heterozygous MTHFR C677T/ A1298C; four pregnant women had heterozygosis C677T in the MTHFR gene. No cases of thrombosis in patients with the mutation in the MTHFR gene A1298C were registered.

Coagulation tests have ruled out natural anticoagulants deficit: the average protein C was 85,81+/- 21,30%; the average level of protein S was 63.10 +/- 13.4%; the average ATIII was 92.06 +/- 8.55%. The determination of D-dimer did not have a great clinical relevance because the values were not significantly altered: the average D-dimer was 728.10 +/- 243.15 µg/L.

All the pregnant women with thrombosis delivered by Caesarean section, in some cases this procedure was scheduled, depending on stage of pregnancy and the hemodynamic stability of the pregnant woman, and, in other cases, the emergency Caesarean section was done because of severe fetal distress induced by the poor hemodynamic status of the mother. Patients diagnosed with antepartum thrombosis were treated with low molecular weight heparin (LMWH) in anticoagulation dose, in two daily subcutaneous (sc) administrations. These patients had Caesarean section under general anesthesia because a period of 24 hours pause for anticoagulation according ASRA [**18**] guidelines for neuraxial anesthesia could not be afforded, even for a single spinal puncture. For patients with cerebral venous or arterial thrombosis, neuraxial anesthesia is absolutely contraindicated.

A period of 24-36 hours without anticoagulant therapy can be risky for a pregnant woman who could suffer recurrent VTE, especially in patients with acute proximal DVT or EP, which occurred in the last month of pregnancy. Such patients benefited from switching sc LMWH on iv unfractionated heparin, which could be discontinued for 4-6 hours before delivery [**19**]. Even such a period of time might be unacceptable for patients with low cardiopulmonary reserve or recent EP. An inferior vena cava filter might be inserted in this situation or the delivery will be done on full-dose anticoagulation [**20**]. After the Caesarean section, the anticoagulant therapy was resumed at 8-12 hours, if the patient had shown no significant perioperative bleeding, with LMWH for 2-7 days, with switching to anti vitamin K therapy by bridging for 72-96 hours with LMWH until the INR of 2-3. The total duration of anticoagulant therapy (pregnancy and postpartum period) was of at least 6 months. Gradual compression stockings at 30-40 mmHg should be considered in women with deep vein thrombosis associated with pregnancy to decrease the risk of long-term postphlebitic syndrome.

## Conclusions

The risk of thrombosis in patients with factor V Leiden is 2.66 times higher than that of the patients negative for this mutation (OR 2.66 95% CI 0.96-7.37 P=0.059). We did not find any statistical association with mutations in the MTHFR gene. Pregnant women with a family history of thrombosis present a 2.18-fold higher risk of thrombosis (OR 2.18 CI 0.9-5.26 P=0.085) (**Fig. 1**). Occurrence of thrombotic events is identified in the last trimester of pregnancy, but especially postpartum. Thrombosis in pregnancy is a redoubtable complication requiring an excellent cooperation between the obstetrician and anesthesiologist.

**Acknowledgments**

This work was supported by the grant PN 42-099 from the Romanian Ministry of Research and Technology.
